# Correlation of Mammography, Ultrasound and Sonoelastographic Findings With Histopathological Diagnosis in Breast Lesions

**DOI:** 10.7759/cureus.32318

**Published:** 2022-12-08

**Authors:** Ruby Thomas, Sudha K Das, Gurumurthy Balasubramanian, Anupama Chandrappa

**Affiliations:** 1 Radiology, Jagadguru Sri Shivarathreeshwara (JSS) Academy of Higher Education and Research, Mysuru, IND

**Keywords:** bi-rads, shear-wave elastography, elastography, breast cancer, ultrasound, mammography

## Abstract

Introduction

Breast masses range from inflammatory and benign to malignant lesions, varying in different age groups and clinical presentations. Breast imaging techniques leading to prompt and specific diagnosis have been a lifesaver for millions around the globe saving them undue mental stress in inflammatory lesions and preventing early death in case of neoplasms. Here, we compare mammography, ultrasonography (US) and ultrasound elastography in the screening and diagnosis of breast lesions and the usefulness of strain ratio as a non-invasive tool in diagnosing breast malignancies.

Aims and objectives

Determining the characteristics of breast lesions on imaging by mammogram, ultrasonography, elastography, calculating strain ratio and correlating them with histopathology. To further estimate the advantages and limitations of one modality over the other in the evaluation of breast lesions.

Methods

The study was done over a duration of 18 months from November 2019 to June 2021 at JSS Medical College and Hospital, Mysuru. In this prospective study, 73 female patients with palpable breast lesions were evaluated using mammography, US and sonoelastography and were co-related with histopathological findings.

Results

This study has proved that the use of ultrasound elastography has higher sensitivity (91.67% with strain ratio kept at 3) in detecting malignant lesions when compared with x-ray mammography and ultrasonography having a sensitivity of 87.88% and 90.91%, respectively. Our study confirmed that there is a correlation between strain ratio and histopathological findings and that the strain ratio has high sensitivity in diagnosing and differentiating malignant breast lesions.

Conclusions

Mammography, US and sonoelastography when combined together are helpful in characterization and management of breast lesions. This helps to avoid unnecessary invasive interventions. The specificity of the study in detecting malignant lesions was comparable with that of histopathological analysis.

## Introduction

Breast masses range from inflammatory and benign to malignant lesions. In Indian women, breast cancer is the commonest, attributing to about 25% of the cancer load [1]. It is the second most common cause of cancer-related mortality [1]. Mammography, B-mode ultrasonography (US) and ultrasound elastography are the most common non-invasive imaging modalities used in identifying breast lesions. Elastography detects pathological tissue alterations in real-time by determining elasticity on compression of tissue under external pressure. The greater the stiffness of the material less is the less elasticity. Benign lesions tend to be softer and less stiff when compared with that of malignant lesions. This property is helpful in differentiating benign and malignant neoplasms [2].

Static and dynamic types are the two types of US elastography. The principle behind this is due to the difference in the type of force applied to deform the lesion. Strain elastography is the major static type while dynamic consists of acoustic radiation force impulse elastography (ARFI) and shear wave elastography. In strain ultrasound elastography, tissue deformation is measured qualitatively by comparing the radiofrequency difference before and after longitudinal compression of the mass [3]. Strain ratio assesses the average strain or relative rigidity in the lesion with that of the surrounding breast tissue with a higher ratio for malignant neoplasms and a lower SR for benign lesions [4]. In this study, we aim to explore, discriminate and characterize between benign breast lesions and malignant breast lesions and to find if US elastography has a superior differentiating role in diagnosing malignant lesions compared to histopathological evaluation.

## Materials and methods

This was a prospective study conducted at JSS Medical College and Hospital, Mysuru after Institutional Review Board approval. Written informed consent was obtained from all the cases prior to enrollment. The study was conducted from November 1, 2019, to June 30, 2021, over a period of 18 months. 73 consecutive female patients with a breast lesion on screening mammography were included in the study. Patients with acute inflammatory conditions of the breast, pregnant and lactating women and those with a previous history of breast biopsy were excluded from the study. All the mammography reporting, ultrasound, and Sono elastography studies were done by a radiologist with experience of over 20 years.

After obtaining a relevant clinical history and written informed consent from the patient, they underwent a mammogram with metal tronica Liliyum mammography system, ultrasonography imaging with real-time elastography using GE Voluson E6 and PHILIPS IU22 machines with a linear probe. Lesions detected by mammography and ultrasound imaging were classified according to the Breast Imaging Recording and Data System (BI-RADS) criteria and BI-RADS Lexicon to one of the 5 categories namely category 1 - negative finding; category 2 - benign finding; category 3 - probably benign; category 4 - finding suspicious for malignancy; category 5 - finding highly suggestive of malignancy. Later, breast elastography was done using a color scale that changes from blue to red in accordance with the strain pattern of the examined tissue.

**Figure 1:**
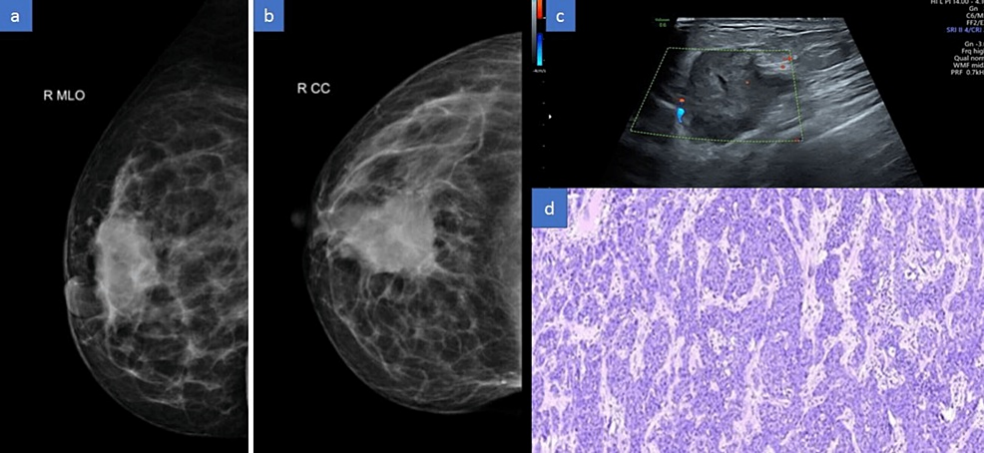
(a-d) A 65-year-old female with palpable mass in the right breast. (a,b) The mammogram (MLO and CC views) shows a large irregular radio-dense mass with irregular and partially indistinct margins. (c) USG B mode image shows an irregularly shaped hetero-echoic lesion with irregular margins and intrinsic vascularity. (d) Histopathology image shows a feature of invasive ductal carcinoma.

**Figure 2:**
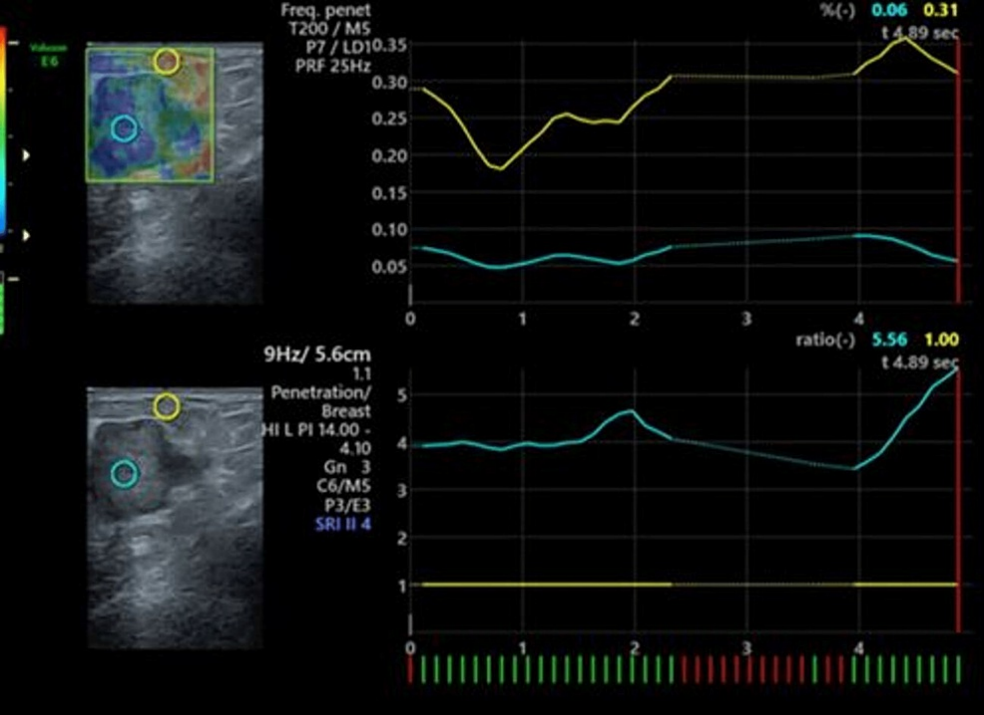
Elastography of invasive ductal carcinoma (same patient images shown in Figure 1). USG elastography showing Tsukuba elasticity score of 4 with high elasticity strain ratio of 5.51

**Figure 3:**
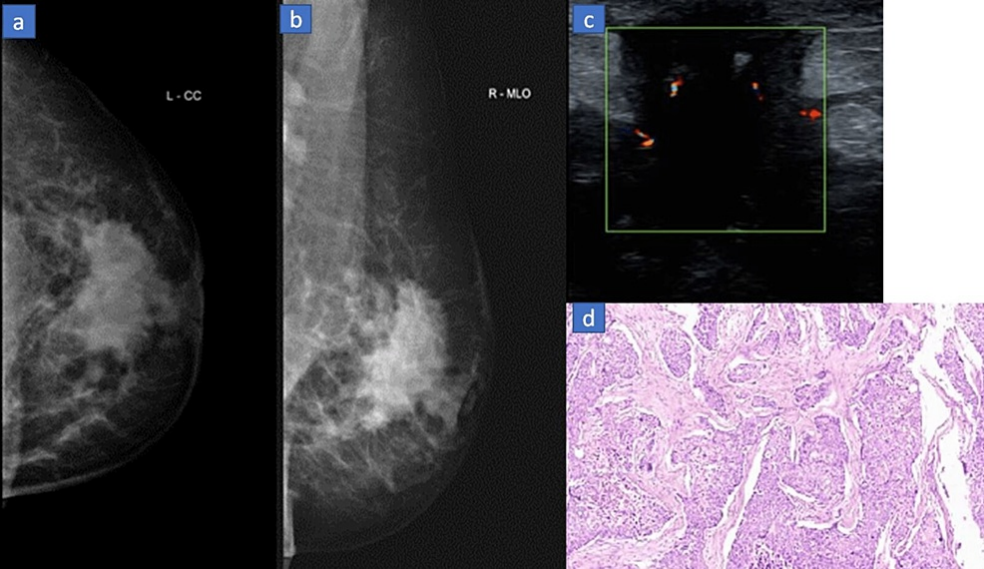
(a-d) A 43-year-old female with a palpable mass in the left breast. (a,b) Mammogram (CC and MLO views) shows a large radio-dense mass with an irregular shape, margins, and few speculations with evidence of adjacent skin thickening and nipple retraction. (c) USG B mode image shows a hypo-echoic lesion with irregular margins and intrinsic vascularity. (d) Histopathology image suggestive of metaplastic carcinoma showing tumor cells arranged in a solid pattern with pleomorphic vesicular nuclei, prominent nucleoli, and moderate cytoplasm. Areas of chondroid differentiation and necrosis with tumor giant cells and lymphoplasmacytic infiltrates were seen.

**Figure 4:**
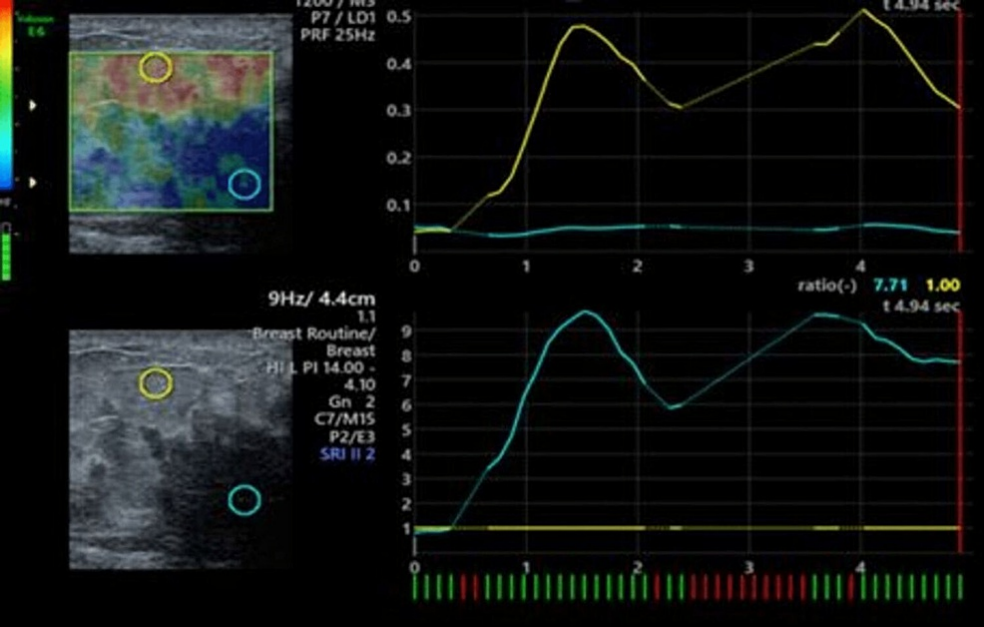
Elastography of metaplastic carcinoma (same patient images as in Figure 3). USG elastography images showing Tsukuba elasticity score of 4 with high elasticity strain ratio of 7.7

**Figure 5:**
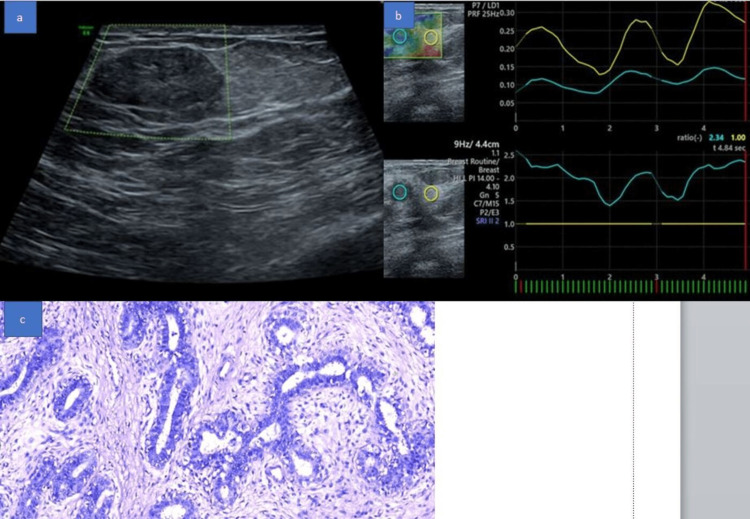
A 41-year-old female with a palpable mass in the left breast. (a) USG B mode image shows a well-circumscribed hypoechoic mass lesion with slightly lobulated margins. (b) USG Elastography images show Tsukuba elasticity score of 2 with high elasticity strain ratio of 2.31. (c) Histopathology image suggestive of fibroadenoma.

Elastographic images were classified according to the 5-score system of Ueno and Itoh et al. [5,6]. The lesion was classified as score 1 for even strain in the entire lesion, as score 2 for strain in most of the lesion with some areas of no strain (mosaic pattern), as score 3 for strain at the periphery of the lesion with sparing of the center, as score 4 for no strain in the entire lesion and as score 5 for no strain in the entire lesion and the surrounding area. Scores 1 to 3 were considered benign while 4 and 5 were accepted as malignant. The real-time elastography strain ratio of breast lesions was calculated using an in-built elastography software. Patients underwent fine need aspiration cytology/biopsy and the HPE results were correlated with the mammography, ultrasonography and real-time elastography imaging findings. Images of invasive ductal carcinoma (Figures 1a-1d, 2), metaplastic carcinoma (Figures 3a-3d, 4) and fibroadenoma (Figure 5) are shown.

 

 

Statistical analysis was done using SPSS 12.0 software for diagnostic sensitivity, specificity, positive predictive value (PPV), and negative predictive value (NPV) of all three techniques and were evaluated by comparing with the histopathologic results. The correlation between elastographic results and histopathologic findings was assessed with the Fisher test and a P-value < 0.05 was considered significant.

## Results

Out of 73 female patients with breast lesions, 40 of them turned out to be benign and 33 were malignant. An increase in age was a risk for the development of malignant lesions. Of the 33 malignant lesions, 25 (more than 75%) were in patients aged more than 50 years. Benign lesions were found mostly in young subjects between the age group 31-50 years. Of the 40 benign lesions, 34 (about 85%) were in the age group of fewer than 50 years. Malignant breast masses presented at a mean age of 57 years with an SD (standard deviation) of 11 years and those having benign lesions had a mean age of 42 years with an SD of 8.

Regarding the side of breast involvement, both breasts were involved in two patients where one lesion was malignant, and another was benign. The left breast was involved in 28 subjects of which 12 were malignant and 16 were benign. The right breast was involved in 43 patients with 23 benign and 20 malignant lesions. Regarding breast composition on mammograms, Type C was the most commonest seen in 38 with 29 being benign and nine being malignant. Type B breast composition had the highest incidence of malignancy with 20 patients having malignant lesions. Type A and Type D breast composition were seen in very few patients. On mammography, the margin of the majority of the benign lesions (95%) was circumscribed. Among malignant lesions four (12.1%) of them had indistinct margins, 20 (60.6%) had spiculated, and nine (27.3%) had micro-lobulated margins.

Calcifications were absent in 35 (85%) of benign lesions and 27 (81.8%) of malignant lesions. Microcalcifications were seen in four (10%) benign and four (12.1%) malignant lesions. Macrocalcifications were seen in two (5%) benign lesions and two (6.1%) malignant lesions. Skin/Nipple retraction was seen in none of the benign lesions but was seen in seven (21.2%) of the malignant lesions. Out of 40 patients with benign lesions, one (2.5%) had skin thickening. Of 33 subjects with malignant breast lesions 6 (18.2%) had skin thickening. Of the 40 benign lesions in our study 14 (35%) had axillary lymphadenopathy and of 33 malignant lesions 32 (97%) had axillary lymphadenopathy. Surrounding architectural distortion was seen in two (5%) of the subjects with benign lesions and 10 (30.3%) of the subjects with malignant lesions.

Of the 40 patients with benign lesions, 34 lesions were having a BI-RADS score of 3, four lesions were having a score of 2 and two of them had a score of 4A. 30 malignant lesions had a BI-RADS score of or more than 4 in x-ray mammogram. Three malignant lesions had a score of 3 on the x-ray mammogram (Tables 1, 2).

**Table 1:** Multivariate analysis of different parameters on mammography

	Benign	Malignant
Breast composition – type		
A	0(0.0%)	3(9.1%)
B	10(25.0%)	20(60.6%)
C	29(72.5%)	9(27.3%)
D	1(2.5%)	1(3.0%)
Shape of the lesion		
Round	7(17.5%)	0(0.0%)
Oval	29(72.5)	0(0.0%)
Lobular	1(2.5%)	18(54.5%)
Irregular	3(7.5%)	15(45.5%)
Margins of the lesion		
Indistinct	2(5.0%)	4(12.1%)
Spiculated	0(0.0%)	20(60.6%)
Microlobulated	0(0.0%)	9(27.3%)
Circumscribed	38(95.0%)	0(0.0%)
Calcifications		
Absent	34(85%)	27(81.8%)
Micro	4(10.0%)	4(12.1%)
Macro	2(5.0%)	2(6.1%)
Skin/nipple retractions		
Absent	40(100%)	26(78.8%)
Present	0(0.0%)	7(21.2%)
Axillary lymphadenopathy		
Absent	26(65.0%)	1(3.0%)
Present	15(35.0%)	32(97.0%)
Architectural distortion		
Absent	38(95.0%)	23(69.7%)
Present	2(5.0%)	10(30.3%)
BI-RADS Mammography		
2	4(10.0%)	0(0.0%)
3	34(85.0%)	4(12.1%)
4A	2(5.0%)	9(27.4%)
4B	0(0.0%)	12(36.3%)
4C	0(0.0%)	1(3.0%)
5	0(0.0%)	7(21.2%)

**Table 2:** Multivariate analysis of different parameters on ultrasonography

	Benign	Malignant
Quadrant wise distribution		
Retro-areolar	3(7.5%)	7(21.2%)
Upper inner	14(35.0%)	3(9.1%)
Lower inner	3(7.5%)	5(15.2%)
Lower outer	9(22.5%)	8(24.2%)
Upper outer	11(27.5%)	8(24.2%)
Multi-focal	0(0.0%)	2(6.1%)
Size of the lesion		
<10mm	1(2.5%)	0(0.0%)
11-20mm	26(65%)	7(21.2%)
>21mm	13(32.5%)	26(78.8%)
Margins of the lesion		
Circumscribed	36(90.0%)	-
Indistinct	4(10.0%)	23(69.7%)
Spiculated	-	10(30.3%)
Echogenicity		
Heteroechoic	5(12.5%)	15(45.5%)
Hypoechoic	30(75.0%)	18(54.5%)
Isoechoic	5(12.5%)	-
Posterior acoustic shadowing		
Absent	40(100%)	19(57.6%)
Present	-	14(42.4%)
Surrounding Architectural distortion		
Absent	39(97.5%)	22(66.7%)
Present	1(2.5%)	11(33.3%)
Vascularity		
Absent	34(85.0%)	2(6.1%)
Present	6(15%)	31(93.9%)
Lymph nodes		
Absent	25(62.5%)	-
Benign	15(37.5)	22(66.7%)
Necrotic	-	11(33.3%)
BI-RADS		
2	2(5.0%)	0(0.0%)
3	35(87.5%)	0(0.0%)
4A	3(7.5%)	5(15.1)
4B	0(0.0%)	9(27.3%)
4C	0(0.0%)	14(42.5%)
5	0(0.0%)	5(15.1%)

Most of the lesions were localized to the upper outer quadrant (19%-26%) followed by lower outer (17%-23.3%) and lower inner quadrants (17%-23.3%). The lower inner quadrant had the least number of lesions. Among the 73, 39 lesions were more than 2 cm. Among benign lesions, 26 were between 11-20 mm and 13 of them were more than 2 cm. Among malignant lesions, most of them were more than 2cm with 26 of them being malignant. Circumscribed margins were present in 36 subjects. All of them were benign. Lesions of 27 subjects had indistinct borders 23 of them turned out to be malignant on HPE. All 10 lesions with speculated margins on ultrasonography turned out to be malignant by HPE. Of the 73, 48 of them were hypoechoic lesions and in them 30 lesions were benign, and 18 lesions were malignant. All five lesions that were isoechoic turned out to be benign on HPE. 20 Lesions were heterochronic of which 15 were malignant on HPE. Posterior acoustic features were absent in benign lesions. Of the 33 malignant lesions 14 had posterior acoustic features. Surrounding architectural distortion was present in one of the 40 benign lesions (2.5%) and 11 out of 33 lesions (33.3%). Out of the 37 subjects whose breast lesions had the presence of vascularity 31 were malignant. Vascularity was absent in 34 benign lesions and 2 malignant lesions.

Necrotic lymph nodes associated with the breast lesion, which are characteristic of malignancy were seen in 11 subjects. All malignant lesions had associated lymph nodes. In BI RADS ultrasound scoring among benign lesions two lesions had a score of 2, 35 lesions had a score of 3, and three lesions had a score of 4A. among malignant lesions 5 had a score of 4A, nine had a score of 4B, 14 had a score of 4C, five lesions had a score of 5. In our study, benign breast lesions had a strain ratio of 2.44 with an SD of 1.27 and malignant lesions were having a mean of 4.93 with an SD of 1.48.

**Figure 6:**
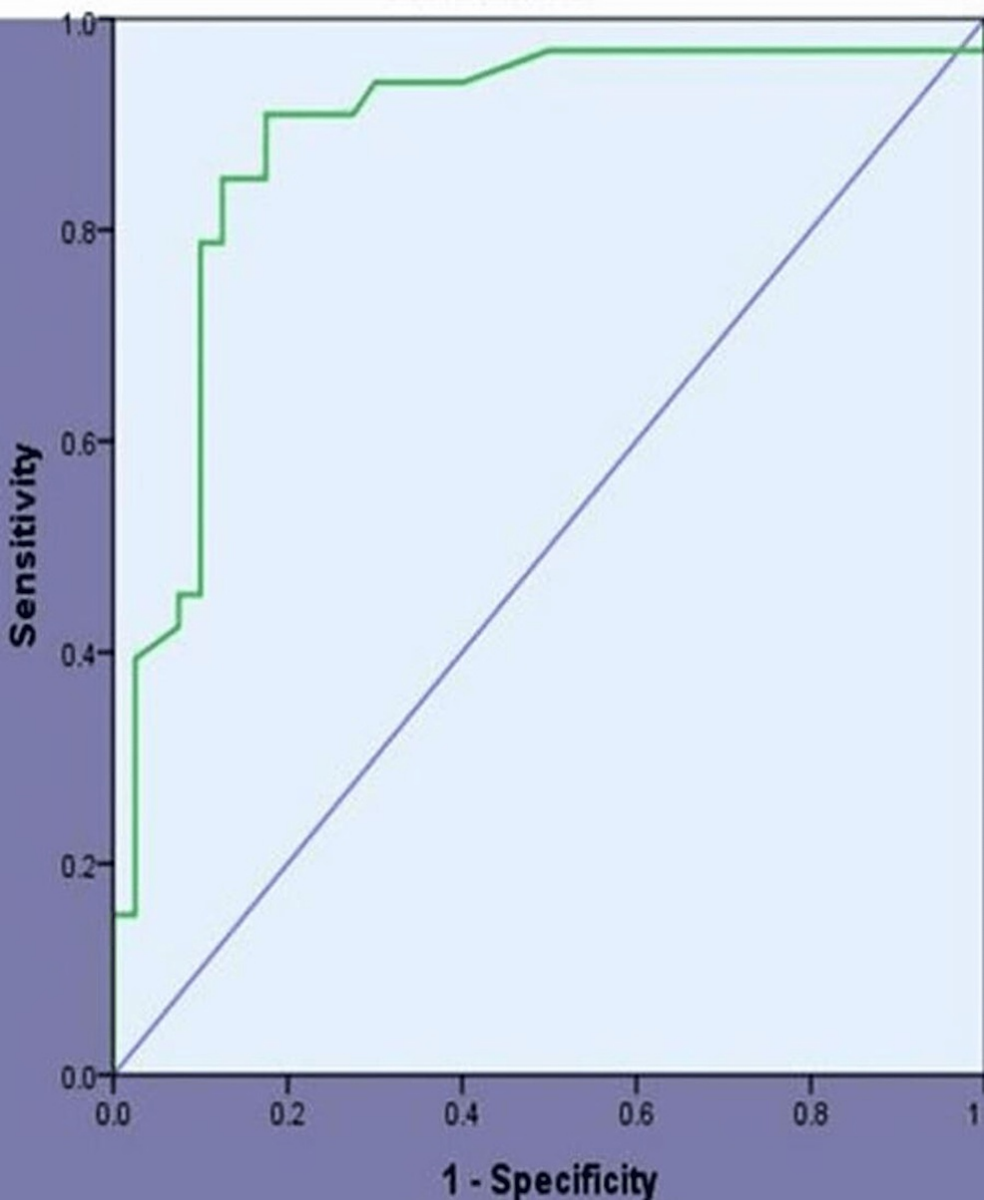
Co-ordinates of the curve in ROC for strain ratio

 In benign lesions, fibroadenoma was the most common benign lesion seen in 32 subjects. The most common malignant lesion was intraductal carcinoma seen in 28 patients (Table 3). Statistical analysis of all the 3 modalities and HPE are shown in Table 4. The diagnostic accuracy of elastography was studied with the ROC curve shown in Figure 6.

**Table 3:** Histopathological diagnosis of the breast lesions

Histopathological diagnosis	Number
Acute suppurative inflammation	2
Benign epithelial lesion	1
Benign proliferative breast disease	2
Chronic granulomatous mastitis	1
Fibroadenoma	32
Fibro adenomatoid hyperplasia	1
Proliferative breast disease with atypia	1
Invasive breast carcinoma	28
Metaplastic breast carcinoma	1
Metastatic adeno carcinoma	1
Lobular breast carcinoma	2
Solid papillary carcinoma	1

 

**Table 4:** Statistical analysis of breast lesions when compared with histopathology

STUDY	MAMMO GRAPHY	ULTRA SONOGRAPHY	US ELASTOGRAPHY SR 2.5	US ELASTOGRAPHY SR 3	US ELASTOGRAPHY SR 3.5
Sensitivity	87.88%	90.91%	86.84%	91.67%	91.67%
Specificity	95.00%	92.50%	100%	100%	100%
PPV	93.55%	90.91%	100%	100%	100%
NPV	90.48%	92.50%	87.5%	92.50%	92.50%
Accuracy	91.78%	91.78%	93.15%	95.89%	95.89%

 

## Discussion

Breast cancer is the commonest malignancy among females in the world. Mammography, B-mode ultrasonography and ultrasound Elastography are the most common methods in clinical practice for identifying breast lesions. In mammography, the incidence of false-negative findings in imaging breast lesions is between 4% and 12% [7]. Due to false negative reports of mammographic imaging in dense breasts, ultrasonogram in dense fibro-glandular breast tissue is also used together. This has considerably reduced the use of invasive methods like biopsy and FNAC based only on mammography findings. Though alone mammograms and the US do not have desirable sensitivity and specificity in diagnosing breast lesions when combined together there is a significant improvement in diagnostic yield and our study results are strongly suggestive of this [8].

In our study, we had 73 patients of different ages comprising 33 malignant and 40 benign lesions on histopathological analysis. We found that the greater the age higher is the risk for the development of malignant lesions. 75.7 % of the malignant lesions were seen in subjects aged more than 50. The lesions described in X-ray mammography were graded according to BI-RADS classification and were compared with HPE. A sensitivity of 87.88 % and Specificity of 95% were found in our study. Sensitivity is comparable with previous studies with Shetty et al. reporting 100% and Barlow et al. 87% [8,9].

In studies by Zonderland et al. and Costantini et al. ultrasonogram features of the benign lesion were a round or oval shape, parallel orientation, margins that are circumscribed, lack of posterior acoustic enhancement, and abrupt interface [10,11]. The echogenic halo, irregular shape, non-parallel, posterior acoustic shadowing, and abnormalities of the surrounding tissue were suggestive of a malignant lesion. In our study, among malignant lesions 69.7% were lobular and 30.3% were irregular in shape. Of the benign lesions, 77.5% were oval in shape. 90% of the benign lesion had circumscribed margins, 69% of the malignant lesions had micro-lobulated margins and 30.3% had spiculated margins. None of the benign lesions had posterior acoustic shadowing features in the US, but 42.4% of the malignant lesions had posterior acoustic features. Surrounding architectural distortion was present in only 33% of the malignant lesions and absent in 97.5% of the benign lesions. The presence of vascularity in the lesion in 93.9% of malignant neoplastic lesions and vascularity was absent in 85% of the benign lesions. These benign lesions turned out to be an atypical benign lesion in HPE. Necrosed lymph nodes were seen in 33% of the malignancies by USG which is a feature highly suggestive of malignancy.

We considered the BI-RADS score of 2 and 3 as benign and the BI-RADS score of 4 and 5 as malignant on both mammography and USG imaging. As shown in the results, mammography had a sensitivity of 87.88% in detecting the malignant lesion and the US had a sensitivity of 90.91%. This is comparable with the studies by Jahan et al. in which sensitivity was 80.0%, and specificity 96.97% by ultrasonography [12]. In another study by Nandan et al., both were respectively 85.4% and 89.31% [13]. Though the credentials of the US as a general screening test are questionable it is more sensitive than mammography in detecting lesions in females with dense breasts. Moss et al. in their studies found that the US along with mammography increased the sensitivity to detect and characterize breast lesions by 14% compared to mammography alone [14].

Elastography when combined with the US based on the firmness of lesions becomes more specific in screening [14]. The color and strain ratio are the parameters helpful in comparing benign and malignant lesions. The strain ratio is an important parameter that helps in the characterization of the lesion by comparing the relative compliance stiffness or deformability of the lesion in comparison with the normal surrounding tissue. Many variations are there among different studies conducted in comparing the strain ratio when a cut-off value for this quantitative method is applied. So, in our study when we kept the strain ratio cut off as 2.5, 3, and 3.5 the sensitivity to differentiate between Benign and Malignant was 86.84%, 91.67%, and 91.67% respectively. In our study, the mean strain ratio for benign lesions was found to be 2.44 out of 40 lesions with a standard deviation (SD) of 1.27 and with regard to malignancy the strain ratio was found to be 4.93 after studying the elastographic properties of 33 lesions with SD of 1.48. In a study by Chhadi et al., the mean strain ratio for benign lesions was 2.2 and the mean strain ratio for malignant was 5.8 [15]. These results were comparable to our study. Zhi et al. in their studies found a cut-off critical value of 3.08 in distinguishing between benign and malignant lesions [16]. Gheonea et al. described a critical value of mean SR of 2.08 for benign lesions and 6.28 for malignant lesions [17].

Elastography alone, when compared with standard US, is less sensitive in imaging lesions that are not focal and has some disadvantages during the evaluation of postoperative changes, diffuse lesions, or large lesions that exceed the probe length or fields of view. If the parenchyma is dense, or fibrous or if there is the presence of hematomas or implants elastography is less sensitive [18].

ROC curve was plotted to quantify the performance of these three imaging methods with the values of sensitivity on the ordinate axis and values of 1- specificity on the abscissa axis. 0.886 was the area under the curve for ultrasound elastography (Figure 6). This is considered a good result. A study conducted by Lee et al. for sonoelastography had a weak performance with the area under the ROC curve of 0.784 [19]. Schaefer et al. had results similar to our study with the area under ROC curve 0.884 for US elastography [20]. In our study, it was proved that combining mammograms, USG, and elastography gives a similar accuracy to that of histopathology in detecting malignant lesions of the breast.

Limitations

Strain elastography has limited usefulness in non-focal and diffuse breast lesions. It is difficult to classify bigger lesions by SE as surrounding healthy tissue will be less. The imaging findings of SE vary with the experience and skill of the examiner. The total cases for the study were relatively small to compare among x-ray mammography, ultrasound, and sonoelastography.

## Conclusions

Imaging by mammography, USG and sonoelastography combined together has higher sensitivity in the differentiation of benign and malignant lesions, hence guiding management. Among the three Sono elastography has higher sensitivity with categorizing lesions as benign and malignant. This helps in avoiding invasive investigations to an extent in breast lesions, by giving them a BI-RADS score and further quantification by strain ratio. The high specificity of these investigations combined together is helpful to rule out potentially malignant lesions and thus help in reassuring patients.

Mammography is not usually carried out in young females due to low sensitivity. In them, USG and US elastography are helpful and efficient alternatives in the characterization of lesions.

The specificity of this study in detecting malignant lesions was comparable with that of histopathological analysis. Thus, it was proved that combining mammograms, USG and elastography gives a similar accuracy to that of FNAC in detecting malignant lesions of the breast.

## References

[1] Sung H, Ferlay J, Siegel RL, Laversanne M, Soerjomataram I, Jemal A, Bray F (2021). Global cancer statistics 2020: GLOBOCAN estimates of incidence and mortality worldwide for 36 cancers in 185 countries. CA Cancer J Clin.

[2] Elkharbotly A, Farouk HM (2015). Ultrasound elastography improves differentiation between benign and malignant breast lumps using B-mode ultrasound and color Doppler. Egyptian J Radiol Nuclear Med.

[3] Bercoff J, Tanter M, Fink M (2004). Supersonic shear imaging: a new technique for soft tissue elasticity mapping. IEEE Trans Ultrason Ferroelectr Freq Control.

[4] Zhao QL, Ruan LT, Zhang H, Yin YM, Duan SX (2012). Diagnosis of solid breast lesions by elastography 5-point score and strain ratio method. Eur J Radiol.

[5] Ueno E, Iboraki P (2004). Clinical application of US elastography in the diagnosis of breast disease. European Congress of Radiology.

[6] Itoh A, Ueno E, Tohno E (2006). Breast disease: clinical application of US elastography for diagnosis. Radiology.

[7] Berg WA, Gutierrez L, NessAiver MS, Carter WB, Bhargavan M, Lewis RS, Ioffe OB (2004). Diagnostic accuracy of mammography, clinical examination, US, and MR imaging in preoperative assessment of breast cancer. Radiology.

[8] Shetty MK, Shah YP, Sharman RS (2003). Prospective evaluation of the value of combined mammographic and sonographic assessment in patients with palpable abnormalities of the breast. J Ultrasound Med.

[9] Barlow WE, Lehman CD, Zheng Y (2002). Performance of diagnostic mammography for women with signs or symptoms of breast cancer. J Natl Cancer Inst.

[10] Zonderland HM, Hermans J, Coerkamp EG (2000). Ultrasound variables and their prognostic value in a population of 1103 patients with 272 breast cancers. Eur Radiol.

[11] Costantini M, Belli P, Ierardi C, Franceschini G, La Torre G, Bonomo L (2007). Solid breast mass characterisation: use of the sonographic BI-RADS classification. Radiol Med.

[12] Jahan AB, Ahmed MU, Begum M (2017). Ultrasonographic evaluation of palpable breast mass and correlation with histopathology. Mymensingh Med J.

[13] Beerappa JR, Balu S, Nandan KLD (2016). Mammographic and sonomammographic evaluation of breast masses with pathological correlation: a prospective original study. Int J Anat Radiol Surgery.

[14] Moss HA, Britton PD, Flower CD, Freeman AH, Lomas DJ, Warren RM (1999). How reliable is modern breast imaging in differentiating benign from malignant breast lesions in the symptomatic population?. Clin Radiol.

[15] Chhadi S, Chhadi T (2018). Ultrasound elastography evaluation of breast masses with FNAC and/or histopathological correlation. Int J Res Med Sci.

[16] Zhi H, Xiao XY, Yang HY, Wen YL, Ou B, Luo BM, Liang BL (2008). Semi-quantitating stiffness of breast solid lesions in ultrasonic elastography. Acad Radiol.

[17] Gheonea IA, Stoica Z, Bondari S (2011). Differential diagnosis of breast lesions using ultrasound elastography. Indian J Radiol Imaging.

[18] Giuseppetti GM, Martegani A, Di Cioccio B, Baldassarre S (2005). Elastosonography in the diagnosis of the nodular breast lesions: preliminary report. Radiol Med.

[19] Lee JH, Kim SH, Kang BJ, Choi JJ, Jeong SH, Yim HW, Song BJ (2011). Role and clinical usefulness of elastography in small breast masses. Acad Radiol.

[20] Schaefer FK, Heer I, Schaefer PJ (2011). Breast ultrasound elastography--results of 193 breast lesions in a prospective study with histopathologic correlation. Eur J Radiol.

